# Epilepsy and associated factors in elderly people of Amirkola, North of Iran (The Amirkola Health and Ageing Project)

**DOI:** 10.22088/cjim.14.1.100

**Published:** 2023

**Authors:** Payam Saadat, Alijan Ahmadi Ahangar, Seyed Reza Hosseini, Ali Bijani, Mohsen Khalili, Shayan Alijanpour

**Affiliations:** 1 Mobility Impairment Research Center, Health Research Institute, Babol University of Medical Sciences, Babol, Iran.; 2 Social Determinants of Health Research Center, Health Research Institute, Babol University of Medical Sciences, Babol, Iran.; 3 Student Research Committee, Babol University of Medical Sciences, Babol, Iran; 4 Students Scientific Research Center, School of Nursing and Midwifery, Tehran University of Medical Sciences, Tehran, Iran; 5 Pre-hospital Emergency, Organization and Emergency Medical Service Center, Babol University of Medical Sciences, Babol, Iran

**Keywords:** Elderly, Epilepsy, Parkinson's disease

## Abstract

**Background:**

Knowledge about the associated factors with epilepsy in the elderly in Iran is limited. Therefore, this study aimed to determine the prevalence of epilepsy and associated factors in Amirkola elderly patients.

**Methods:**

This cross-sectional study is a part of a comprehensive and cohort research of "The Amirkola Health and Ageing Project". The Mini-mental State Examination was used for cognitive impairment, Geriatric Depression Scale for psychiatric diseases and the Physical Activity Scale for Elderly questionnaire for physical activity.

**Results:**

The prevalence of epilepsy was 35 from 1482 participants (24/1000). The significant association between Parkinson’s Disease (OR=6.25, 95%CI=1.35-28.4, P=0.001), falls (OR= 3.81, 95%CI=1.62-8.97, P=0.001), depression (P=0.001), hyperphosphatemia (P=0.039) and hypokalemia (P= 0.031) concluded with epilepsy. Past history of stroke (6 % versus 2%, OR= 2.8, 95%CI, 0.97-8.27, P=0.07), increased serum level of triglyceride (OR= 1.96, 95%CI= 0.99-3.88, P=0.06) and low-density lipoprotein (LDL) (P=0. 45) were seen in epileptic patients vs. non-epileptic patients.

**Conclusion:**

Parkinson's disease, frequency of falls and depression were the associated factors in epileptic patients and a correlation between past history of stroke, increased serum level of triglyceride and LDL with epilepsy were seen. Associated factors required screening, diagnosis and treatment.

Epilepsy is one of the most common serious brain conditions, affecting over 70 million people worldwide (1), with a lifetime prevalence of around 8 per 1000 individuals worldwide. The Global Burden of Disease Study estimates epilepsy to be among the 30 leading causes of years of life lost due to disability(2). In recent decades, there has been a growing interest in epilepsy in elderly patients(3). Due to the increasing population of older adults in different societies, the incidence of epilepsy increases as the elderly people age (4). The known underlying causes of epilepsy and seizure that trigger aging strokes, dementia, brain tumors, head trauma, and infectious diseases such as meningitis or encephalitis (5). Toxins and the complications of drugs used in these ages and herbal medicines, metabolic problems, such as hypoglycemia and hypocalcemia, can also be considered as accelerating epileptic episodes in this age (6). There is no consensus on whether the association of factors such as cognitive impairment or psychiatric disorders in epilepsy of elderly people can be considered as an etiologic factor or its complications An atypical and uncommon clinical manifestation of epilepsy in the elderly causes the diagnosis of epileptic seizures difficult in the older people (7). 

With this in mind, the diagnosis of epileptic seizures in the elderly should be more precise to justify the initiation of anti-epileptic drugs. In studies on the causes of epileptic seizures in the elderly, about one-third to half of the cases remain without etiology(8). Research on elderly epilepsy has become more important in recent years. By identifying the causes and factors related to epilepsy and how to prevent them, it is possible to reduce the incidence of epilepsy and complications caused by these disorders (such as fractures), and most importantly, to reduce the mortality rate of the elderly due to the disease. Considering all the above, the aim of this study was to determine the prevalence of epilepsy in the elderly and the associated factors in the elderly population of Amirkola.

## Methods

This cross-sectional study is a part of a comprehensive and cohort research of "The Amirkola Health and Ageing Project" (AHAP)(9). The project began in 2011-2012 in Amirkola, a town located near the Caspian Sea, in northern Iran and is still ongoing. Amirkola is a small and middle-class income town with a low immigration rate and a suitable model of a town for cohort studies. It has been known as the first comprehensive cohort study of the health of older people ever conducted in Iran. Amirkola cohort study was designed with regard to multiple chronic diseases and health concerns like as osteoporosis, cognitive impairment and falls. The strengths of this cohort project are the different exposures and the adequate coverage of the population of which more than 70% of the elderly aged 60 years and over in Amirkola were entered in this cohort (10). 

This study was approved by the Ethics Committee of Babol University of Medical Sciences (MUBABOL.HRI.REC.1395.130). A written consent was received from each participant before data collection phase. In this study, 1482 elderly people (60 years and above) were enrolled and whose information is available at the Social Determinants of Health Research Center (Babol University of Medical Sciences). By using a census sampling, the elderly population was divided into three age groups: 60-69 years, 70 to 79 years, and 80 years and over. To collect the information needed for this study, as the first questionnaire was completed by the participants or if necessary, by their companions. This questionnaire contains demographic questions that include age, gender, education, occupation, marital status, and medical history which is collected self-declaration.

For illiterate patients, all questions were read by the interviewer and tried to resolve any ambiguities in understanding the questions. The first stage of the AHAP is evaluating and recording weight, height, blood pressure (BP), and body mass index (BMI), according to the findings of the questionnaire and the medical records of the trained personnel. The diagnosis of epilepsy in the elderly and the onset of it and the use of the anti-epileptic drug in this study were explanatory in all studied cases confirmed by the neurologist responsible for this study. 

History of stroke, ischemic heart disease (IHD), head trauma, dementia, brain tumors and other progressive neurodegenerative disorder such as Parkinson’s disease(11, 12) were filled in a questionnaire. For cognitive impairment we used the Mini-mental Examination (MMSE). Also, psychiatric diseases were assessed by Geriatric Depression Scale GDS. Furthermore, physical activity was measured based on the standard physical activity questionnaire for the elderly; the Physical Activity Scale for Elderly (PASE). Finally, specialists in these diseases in AHAP confirm these cases.

The elderly’s weight was measured in participants wearing light clothes. Body mass index was measured and recorded according to the height and weight of the patients. Those elderlies with BMI of 25 to 29 are considered normal weight, the overweight older people are those with BMI ≥30 and elderlies with BMI <25 were considered as weight loss. Complete blood cell (CBC), serum levels of vitamin D, zinc, copper, phosphorus, calcium, sodium and potassium were assessed. The exact criteria for defining the diseases, associated factors, the questionnaires and normal serum level of laboratory tests at the AHAP project were available and accessible (10).

The criteria for entering the study were elderly people diagnosed with epilepsy previously or taking antiepileptic drugs. Exclusion criteria were; syncope, fall, confessionals states, and other differential diagnosis of epileptic seizures, metabolic disorders such as hypoglycemia, transient global amnesia, migraine attacks with aura, transient ischemic attacks (TIA) and sleep disorders (parasomnia). Alcohol consumption and sleep deprivation (provocative seizure factors) were excluded as a cause of epilepsy.

**Statistical analysis: **For statistical analysis, the elderly people were divided into two main groups; the first group included patients who had been diagnosed with epilepsy and the second group was not epileptic, but were residents in Amirkola town and entered in the AHAP. Collected data were entered into the software SPSS (23), and statistical analysis was performed. Chi-square and t-tests were used to evaluate the association between related disorders with epilepsy. Using logistic regression analysis, other variables affecting epilepsy were controlled. A p˂0.05 indicated a meaningful level.

## Results

Of 1482 elderly, 35 participants were epileptic. Hence, the prevalence of epilepsy was 35 from 1482 participants (24/1000) (OR=2.36, 95%CI=1.58-3.13). The distribution of gender was 825 (55.7%) males vs. 657(44.3%) females, although most of the 21(60%) epileptic patients were females, the frequency of the female was lower than the male in the studied elderly people, frequency of epilepsy in elderly women was non significantly higher than elderly men (P=0.08). Table 1 shows the characteristics of elderly patients with and without epilepsy. 

The distribution of demographic and clinical characteristics and their associations with epilepsy in elderly individuals by the calculation of odds ratio (OR) with a 95% confidence interval (95%CI) are demonstrated in table 2. The mean age of epileptic elderlies based on table 1 was (67.5±7.5). The difference between the mean age of epileptic and non-epileptic elderly patients (69.1±7.2) was not significant while the frequency of epilepsy in elderly age more than 70 was higher than the group less than 70 years non-significantly (P=0.20). Sixty-seven elderly people had history of stroke of which 6% had epilepsy, in other words 11% of epileptic elderly had history of stroke. Although the proportion of epilepsy in the participants with history of stroke was higher than those without stroke, but the difference was not statistically significant (6 % versus 2%, OR=2.8, 95%CI, 0.97-8.27, P=0.07). 

The proportions of epilepsy in the elderly, with and without Parkinson’s disease were 12.5% and 2.3%, respectively (table 2), indicating a significant association between Parkinson’s disease and epilepsy (OR=6.25, 95%CI=1.35-28.4, P=0.001). Similarly, there was a positive association between history of falls and depression with epilepsy. Falls occurred in 20% of epileptic patients versus 6.2% of those without epilepsy (OR= 3.81, 95%CI=1.62-8.97, P=0.001). Moreover, the occurrence of epilepsy in depressed elderly was twice higher in non-depressed people (3.7% vs. 1.4%), (OR= 2.68, 95%CI=1.32-5.43, P=0.001).

**Table 1:** Characteristics of elderly people with and without epilepsy (n=1482)

**Variables**	**Epilepsy(+)**	**Epilepsy (-)**	P-value*
Age (years)	67.5+7.5	69.1+ 7.2	0.20
BMI (kgm^2^)	27.33+5.5	27.17 +4.5	0.84
FBS (mg/dl)	116.0+48.3	118.7+46.0	0.73
Cholesterol (mg/dl)	205.9+53.4	196.4+42.0	0.19
Triglycerides (mg/dl)	188.2+103.6	160.2 +84.1	0.054
HDL-C (mg/dl)	38.2+4.5	38.7+4.4	0.45
LDL-C (mg/dl)	138.6+35.3	126.4+36.3	0.057
Calcium (mg/ml)	9.28+0 .56	9.25 + 0.43	0.68
Phosphor (mg/ml)	4.14 + 0 .65	3.92 +0.61	0.039
Na (mg/ml)	140.1+2.08	140.1+10.8	0.63
K (mg/ml)	4.09+0.27	4.19+0.28	0.031
Ferritin (ng/ml)	167.5+108.8	164.6+125.7	0.89
Serum vitamin D (ng/ml)	35.2 +34.1	34.1+31.8	0.83
SBP (mm Hg)	140.5+23.3	142.7+22.1	0.55
DBP (mmHg)	82.4+13.0	81.4 +11.8	0.64
GDS	6.28+3.74	4.45 +3.42	0.58
Comorbidities	4.85+2.0	2.65 +1.9	0.32
MMSE	25.1+3.9	25.4+3.5	0.66
Total physical activity^#^	104.7 + 48.4	108.1 + 61.6	0.76

Note. BMI, body mass index; HDL-C, high density lipoprotein; FBS, fasting blood sugar; LDL-C, low density lipoprotein; Na, sodium; K, potassium; SBP, systolic blood pressure; DBP, diastolic blood pressure; GDS, Geriatric Depression Scale; MMSE, Mini Mental State Examination. * Compared by independent t-test # Determined by using questionnaire of daily physical activity in the elderly

As shown in table 1 , serum levels of phosphorous were significantly higher in epileptic patients compared to healthy people (P=0.039) and also the serum levels of potassium were significantly lower compared to healthy people (P= 0.031).In addition , there was a trend towards a higher level of serum triglyceride in epileptic patients compared to healthy people (P=0.054) .On the other hand, the proportions of epilepsy in the elderly, with and without hypertriglyceridemia were (3.2% vs. 1.7%), indicating a non-significant association between the higher level of serum triglycerides and epilepsy OR= 1.96, 95%CI= 0.99-3.88,P=0.06 .

**Table 2:** The associations between demographic and clinical characteristics with epilepsy in the elderly people

**Variables**	**Epilepsy (+)** **n (%)**	**Epilepsy (-)** **n (%)**	**Odds ratio** **(95%, CI)**	**P-value**
Age (years)				
< 70	24(2.7 )	835(97.3)	1.0 (Ref)	
> 70	11(1.7)	612(98.3)	1.6(0.78-3.29)	0.20
Sex				
Male	14 (1.7)	811(98.3)	1.0 (Ref)	
Female	21 (3.2)	636(96.8)	1.91 (0.96-3.79)	0.08
Educational levels				
Illiterate	22(2.3)	919(97.7)	1.0 (Ref)	
Educated BMI, kg/m2	13(3.0)	528(97.6)	0.97(0.49-1.95)	0.93
25-30	6(0.9)	632(99.1)	1.0 (Ref)	
<25	18(3.7)	468(96.3)	4.05(1.59-10.28)	0.003
> 30	11(3.1)	347(96.9)	3.33(1.22-9.10)	0.019
Hypertriglyceridemia				
No < 150 mg/dl	14(1.7)	820(98.3)	1.0 (Ref)	
Yes > 150 mg/dl	21(3.2)	627(96.8)	1.96(0.99-3.88)	0.06
Abnormal HDL-C value				
No	3 (1.1)	265 (98.9)	1.0 (Ref)	
Yes	32 (2.6)	1182 (97.4)	2.39 (0.72-7.86	0.18
Marital status				
Single	30 (2.4)	1234 (97.6)	1.0 (Ref)	
Married	5(2.3)	213 (97.7)	0.96 (0.37-2.51)	1.0
History of stroke				
No	31 (2.2)	1384 (97.8)	1.0 (Ref)	
Yes	4 (6.0)	63 (94.0)	2.83 (0.97-8.27)	0.07
MetS #				
No	7 (1.9)	366 (98.1)	1.0 (Ref)	
Yes	28(2.5)	1081 (97.5)	1.35(0.58-3.12)	0.55
Diabetes				
No	26 (2.5)	1000 (97.5)	1.0 (Ref)	
Yes	9(2.0)	447 (98.0)	0.77(0.36-1.66)	0.51
Hypertension¥				
No	13 (2.3)	548 (97.7)	1.0 (Ref)	
Yes	22(2.4)	899 (97.6)	1.03(0.51-2.06)	0.93
History of CVD				
No	28 (2.5)	1110 (97.5)	1.0 (Ref)	
Yes	7 (2.0)	337 (98.0)	0.82 (0.35-1.90)	0.64
History of falls				
No	89 (6.2)	1358 (93.8)	1.0 (Ref)	
Yes	7(20.0)	28 (80.0)	3.81(1.62-8.97)	0.001
Parkinson disease				
No	33 (2.3)	1433 (97.7)	1.0 (Ref)	
Yes	2(12.5)	14(87.5)	6.20(1.35-28.39)	0.001
Smoking				
No	29 (2.4)	1172 (97.6)	1.0 (Ref)	
Yes	6 (2.1)	275 (97.9)	0.88 (0.36-2.14)	1.00
Depression				
No	12 (1.4)	844 (98.6)	1.0 (Ref)	
Yes	23 (3.7)	603 (96.3)	2.68 (1.32-5.43)	0.001

Note. BMI, body mass index; HDL-C, high density lipoprotein; MetS, metabolic syndrome; CVD, cardiovascular disease.

* Using chi square test

# Diagnosed according to Iran criteria

¥ Defined as systolic blood pressure > 140 mm/Hg and/or diastolic blood pressure > 90 mm/Hg

Similarly, there was a non-significantly higher serum level of LDL in epileptic elderlies compared to healthy people (P=0.057), while there was not any significant association between the serum level of HDL in the two studied groups (P=0.45). The difference between the BMI of epileptic and non-epileptic elderly patients was not significant (P=0.84), while as shown in table 2, the prevalence of epilepsy was higher in lower BMI elderly people (P=0.003) and so on obese subjects (P=0.019) compared with normal weight elderly. 

According to the data, epilepsy prevalence decreased with educational level progression but it was not significant (P=0.93). By considering the other data, patients with and without epilepsy were similar regarding demographic features like marital status, total physical activity, MMSE, also there was no statistically significant association between epilepsy in the elderly with cardiovascular disease, hypertension, metabolic syndrome, diabetes mellitus, smoking, and several other measured biochemical characteristics and variables.

## Discussion

In this study, the prevalence of epilepsy in elderly people was 2.4%. In a study among the US Medicare beneficiaries, the average annual prevalence and incidence rates were 10.8/1,000 and 2.4/1,000(13). In other studies, different incidence was reported so as in the study by Giussan et al., the prevalence of epilepsy was 4.57 per 1,000. The incidence was 53.41 per hundred thousand and the highest incidence of epilepsy was among the elderly(14). In a study on epilepsy in Finland, the incidence of epilepsy in the elderly has increased by 3.5% per year(15). In other studies, there are reports of varying rates of incidence and prevalence of epilepsy in the elderly, but overall, these rates are higher in the elderly than in any other periods of life. 

Also in our study, the prevalence of epilepsy increased with age, while the mean age of epileptic elderly (67.5±7.5) was less than previous studies in this field(4). Perhaps one of the reasons for lower age onset of epilepsy in the elderly in this area may be due to lower age onset of stroke, which is the common cause of epileptic seizures in older adults. In other similar studies, the incidence ranges from 1 to 3 per 1,000 person-years in people with epilepsy, and the incidence rate of this study is in the average of these rates. It should be concluded that the indicators for health care status of the elderly population are generally acceptable in this area. The prevalence of epilepsy in our study elderly population with history of stroke was 6%, while 11% of epileptic elderly had history of stroke. However, the proportion of epilepsy in older adults with history of stroke was higher than those without history of stroke, although the difference did not reach a statistical significant level in most other studies, stroke is the common cause of seizures in the elderly population(16). In a retrospective cohort study(17), the diagnosis of epilepsy was given to a total of 10843 elderly people for the first time. In these older adults, the common causes of epilepsy were: strokes, dementia, brain tumors and other central nervous system diseases. Similar results have been reported in other studies with regard to the incidence of post stroke seizures in the elderly(18). 

In a previous study in Babol (a city nearby Amirkola) in northern Iran(19), the incidence of seizure in 250 cases of stroke was 42 (17.3%) during the two-year follow-up. In 14 seizure cases, the episodes were repeated. Finally, in this study, (5.6%) cases of stroke suffered from epilepsy that was similar to findings of this study. In the epidemiological study of elderly epilepsy in Iran, it was reported that 24% of the causes of elderly epilepsy were related to stroke(20). Given that stroke is a predominantly "elderly illness" and also because strokes are somewhat preventable, engaging in preventive measures to reduce the incidence of stroke, definitely can reduce complications caused by stroke (21) such as epilepsy in elderly people. Another important finding of this study was the significant association between Parkinson's disease and epilepsy in the elderly of this area of investigation. In comparison with other studies in this field, the amount of this companion has been higher in current study. Some researchers believe that Parkinson's disease associated with epilepsy in the elderly is more likely due to the combination of comorbid brain disorders with P.D (22), while in several reports point to the problem of misdiagnosis of non-motor epilepsy with non-motor manifestations of P.D. (23) and others point to low rate of this association, the cause of this association is attributed to the abnormal intra cortical excitability in P.D (24).

In other reports, it has been concluded that although there is no direct correlation between Parkinson's disease and epilepsy in the elderly pathological changes found in the P. D patient's brain including cortical thinning and architectural changes may be epileptogenic(25). Nonetheless, it seems some justifications are necessary for the causes of this significant association with the current study, in particular if these findings are confirmed in later studies in other areas. According to the findings of this study, the frequency of falls in aging epileptic people was triple the rate compared to non- epileptic cases. Although seizure attacks may lead to falls, some acute seizures may occur following a traumatic brain injury due to falling. Another cause of the fall in the elderly can be the side effects of taking some medications. The difficulty of diagnosing epilepsy in the elderly (26) also leads to this association between falls and seizures which is poorly documented.

Nevertheless, there are reports that a genetic basis (ApoE4 allele) is related to the risk of posttraumatic epilepsy (27), but in practice, the older adults suffer more likely the risk of acute seizure following a traumatic brain injury than young adults (28). It is clear that preventing and treating each one can reduce other occurrences(29). The findings of this study showed that the frequency of epilepsy in elderly patients with depression was twice as high more than those elderly without depression. In Ettinger et al.’s study, it was shown that psychiatric disorders such as depression, anxiety, psychosis and substance abuse in elderly patients who were referred for the new-onset epilepsy were more than the control group (30). In Saadat et al.’s study, anxiety and depression were the most common symptoms in epileptic patients(31). Other studies disclosed that depression is the most common comorbid psychiatric disorder in patients with epilepsy(32), and the lifetime prevalence of depression in association with epilepsy is as high as 55%.(33). Knowledge of the relationship between depression and epilepsy in the elderly is important. This association is also a two-way, of which preventive or therapeutic measures for each of them can have a positive effect on the others. Yet epilepsy treatment is usually done, but due to fear of exacerbation of epileptic seizure attacks due to side-effects of antidepressant drugs, treatment for depression is less done in elderly epileptics. Certainly, untreated depression in different ways can exacerbate epileptic seizures and reduce the quality of life and even increase the mortality rate of these patients, 

Cognitive impairment in our study did not correlate significantly with the occurrence of epilepsy in the elderly, but the mean score of MMSE in epileptic elderlies was lower than the non-epileptics. Most studies showed that in cases of cognitive impairment, the occurrence of epileptic seizures had increased (34).There was a trend towards a higher level of serum triglycerides in the elderly epileptic patients compared to healthy people. In other studies, hypertriglyceridemia as a dyslipidemia in epileptic patients was reported(35). The cause of dyslipidemia in this study was attributed to using enzyme‐inducer anti-epileptic drugs.

Given the high levels of triglyceride and LDL (known risk factors of atherosclerosis) in the current study, can these risk factors play a role in the development of epilepsy in the elderly, a question that needs to be answered.

One of the findings of this study was the trend of lower level of education in elderly epileptics compared with non-epileptics. In many studies(35), the same results were obtained. However, educated people are more aware of the disease and the ways to prevent it. Although the lower occurrence of epilepsy was seen in our study elderly population with normal weight, the occurrence of epilepsy was higher in low BMI and obese elderly. Results from other studies in this field are similar to our findings(36). With these findings, to have a good weight has been advised to the elderly to prevent this disease, but also, in many other diseases in this era of life. In clinical daily practice, electrolyte disturbances such as (hyponatremia, hypocalcemia, and hypomagnesemia) can manifest with seizures(37), but our findings of hyperphosphatemia and hypokalemia being more in elderly epileptics compared to healthy elderly people cannot be justified. In the event of getting similar results from other studies in this field, there should be an underlying cause for these findings. Ultimately, these findings may be helpful in preventing or treating these patients.

 Despite the current study was a community-based study as a strong point, but there were limitations. One of the limitations of this study was the small sample size of elderly epileptics, which made it difficult to compare the relevant variables in the epileptic and non-epileptic elderly groups. Failure to distinguish the clinical epileptic syndromes such as Complex Partial Seizures (CPS) from other epileptic seizure syndromes was one of the other limitations of this study leading to not differentiating the types of epileptic seizures attacks. In addition, in the present study, the types of strokes had not been determined which became a limitation of this study.

In conclusion parkinson's disease, frequency of fall and depression were the associated factors in epileptic patients and correlation of past history of stroke, increased serum level of triglyceride and LDL with epilepsy were seen. Lack of association between cognitive impairment with occurrence of elderly epilepsy was another finding of the study. A trend towards a higher level of serum triglycerides and LDL was observed in epileptic elderlies. We cannot justify the high serum levels of phosphorus and low serum levels of potassium in these elderly epileptics. Associated factors required screening, diagnosis and treatment. Further studies are needed to investigate the probability of other associated factors.
